# Comparison of peripheral venous and arterial blood gas in management of patients with respiratory complaints in the emergency department: A prospective observational cohort study

**DOI:** 10.1371/journal.pone.0330190

**Published:** 2025-09-05

**Authors:** Sarah Körver, Maud B. R. C. Eurlings, Audrey H.H. Merry, Michiel H.M. Gronenschild, Maarten T.M. Raijmakers, Gideon H. P. Latten

**Affiliations:** 1 Emergency Department, Zuyderland Medical Center, Heerlen, The Netherlands; 2 Department of Epidemiology, Zuyderland Medical Center, Heerlen, The Netherlands; 3 Department of Pulmonology, Zuyderland Medical Center, Heerlen, The Netherlands; 4 Department of Clinical Chemistry, Zuyderland Medical Center, Heerlen, The Netherlands; Universitair Kinderziekenhuis Koningin Fabiola: Hopital Universitaire des Enfants Reine Fabiola, BELGIUM

## Abstract

**Introduction:**

Although peripheral venous blood gas (pVBG) analysis is used in the Emergency Department (ED), its effect on clinical decision making is unknown. We assessed whether pVBG analysis combined with pulse oximetry could replace arterial blood gas (ABG) analysis to determine treatment and disposition of ED patients with respiratory complaints. In addition, we assessed agreement between venous and arterial values and pulse oximetry (SpO_2_).

**Method:**

We performed a 12-week prospective observational study in ED patients with respiratory complaints. ABG and pVBG samples were drawn as simultaneously as possible, with a maximum of five minutes in between. Physicians initially determined treatment and disposition using pVBG results, after which they were shown the ABG results. Subsequent alterations in treatment and disposition were registered. We calculated pVBG and ABG mean differences (MDs) using Bland-Altman analysis and SaO_2_ and SpO_2_ MD and correlation using Passing-Bablok regression analysis and Bland-Altman analysis.

**Results:**

In 56/154 (36.4%) patients, the ABG results changed the preliminary treatment and disposition. Most (57.5%) changes consisted of a change in supplemental oxygen therapy. The MDs (95% CIs) between pVBG and ABG results were: pH −0.04 (−0.05 to −0.04) pH units, bicarbonate 1.57 (1.20 to 1.93) mmol/l, pCO_2_ 0.85 (0.70 to 0.99) kPa and lactate 0.34 (0.28 to 0.40) mmol/l. We found a good correlation between the SaO_2_ and SpO_2_.

**Conclusion:**

In over one third of patients with respiratory complaints in the ED, ABG results changed treatment and/or disposition based on pVBG results. Most changes could be considered as minor. The arterial pO_2_ was most frequently mentioned as the reason for the changes.

## Introduction

Blood gas analysis plays an important role in the work-up of many emergency department (ED) patients. Arterial blood gas (ABG) analysis is considered the reference standard test for evaluating the respiratory and metabolic state of (critically) ill patients, but is associated with drawbacks. First, the puncture is painful [1,2] and can only be performed by qualified nurses or physicians. Second, complications can occur, of which post-puncture hematomas are most common [3]. Rarely, more serious complications include arterial dissection and digital ischemia [4].

Over the last years, several authors have suggested that a peripheral venous blood gas (pVBG) could be used as an alternative to ABG in the ED [3,5–7]. Studies have already shown good correlation and agreement between arterial and venous pH and bicarbonate in several patient populations, among which diabetic ketoacidosis (DKA), acute exacerbation of chronic obstructive pulmonary disease (COPD) and patients with sepsis [1,3,5,7–14]. Although agreement between arterial and venous pO_2_, pCO_2_ and lactate is insufficient, pVBG analysis can exclude arterial hypercapnia and hyperlactatemia, by using specific cut-off values [9,15–17]. In addition, professionals could use peripheral pulse oximetry as a reliable non-invasive alternative to estimate the arterial oxygen saturation (SaO_2_) [18–20].

Despite these recommendations, ABG is still part of standard practice in patients with respiratory complaints, which may be due to the lack of literature on clinical decision making. It is unknown whether treatment, disposition and clinical outcomes are different when based on either ABG or pVBG analysis. Since respiratory complaints occur in a significant proportion of ED patients, streamlining care is desirable, especially in times of widespread ED crowding.

In this study, we aimed to assess whether pVBG analysis combined with pulse oximetry could replace ABG analysis to determine treatment and disposition of patients with undifferentiated respiratory complaints in the ED. In addition, we aimed to assess the agreement between venous and arterial blood gas values and peripheral oxygen saturation.

## Methods

### Design and setting

We performed this prospective observational single-centre cohort study during a 12-week period between 21 October 2019 and 13 January 2020 at Zuyderland Medical Center, a large teaching hospital located in Heerlen, the Netherlands. The study was reviewed and approved by the local medical ethics committee (METC-Z2019070).

### Study population

Eligible for inclusion were adult patients (≥18 years) with respiratory complaints, a reliable peripheral oxygen saturation measured by pulse oximetry (Philips IntelliVue MP30) and an indication for an ABG (as determined by the treating physician conform standard practice). We defined ‘respiratory complaints’ as a subjective feeling of dyspnoea, a respiratory rate >20/minute, or a peripheral oxygen saturation <95% with or without supplemental oxygen therapy. Exclusion criteria were inability to consent to study participation, ABG analysis only required for other reasons (e.g., electrolytes analysis) and previous participation in the study.

We calculated the required sample size using the formula to estimate a proportion or apparent prevalence with specified precision. Aiming to detect alterations in treatment in 1:10 patients with a confidence interval of 95%, at least 139 participants were required, to which we added 10% for possible dropouts. The total required sample size was 153.

### Informed consent and data collection

All eligible patients were approached for participation upon arrival in the ED and received standard care. After giving verbal consent to the treating physician to study participation the ABG and pVBG samples were collected. As soon as possible after initial treatment and stabilization, the patient or their representative received additional verbal and written information. Subsequently, written informed consent was obtained within 24 hours. Only after the written informed consent were data registered in the study database. If the patient died before giving written consent, the collected data were included in the study and the representative was informed of inclusion in the study [21].

ABG and pVBG samples were drawn as simultaneously as possible, with a maximum of five minutes in between. During that period, no change in therapy was initiated and no additional tests were performed. When possible, pVBG samples were drawn without the use of a tourniquet. Immediately after sampling, blood gas analysis on both venous and arterial blood was performed on the RAPIDPoint 500 Blood Gas System (Siemens-Healthineers, The Hague, The Netherlands), located within the ED itself. The treating physician subsequently only received the pVBG results, after which preliminary decisions on treatment and disposition were registered. Next, the treating physician received the ABG results, after which he/she filled out a 3-question survey to determine whether an alteration in treatment or disposition was necessary, based on the ABG results instead of the pVBG results. Definitive treatment and disposition were at the physician’s discretion.

Patient data were collected using a digital Case Report Form (CRF) created with the Research Manager Data Management module (Research Manager, Deventer, the Netherlands). For each patient, we registered: age, gender, comorbidities (Charlson Comorbidity Index (CCI) [22]), smoking status (never, current or ex-smokers), recent hospital admissions for similar complaints or diagnosis (<30 days), vital signs measured at the time of blood gas sampling (blood pressure, pulse rate, oxygen saturation, respiratory rate, temperature and mental status), disposition, duration of hospital admission, treatment with mechanical ventilation, diagnosis at hospital discharge (either from the ED or after admission), in-hospital mortality and treating physician in the ED (name and specialty).

### Study endpoints

The primary study endpoint was the frequency with which treatment and disposition were altered, based on ABG results, when compared to using pVBG results only.

The secondary endpoints were the types of alterations, the parameters of the ABG causing the alterations, the agreement between pheripheral venous and arterial pH, bicarbonate, pCO_2_, lactate and pO_2_ and the correlation and the agreement between the SaO_2_ and the oxygen saturation measured with pulse oximetry (SpO_2_).

Treatment alterations were divided into five subgroups: (1) alteration of disposition, (2) alteration of supplemental oxygen therapy, (3) alteration of mechanical ventilation, (4) performance of additional tests, and (5) other. We considered changes in disposition and changes in mechanical ventilation as major alterations, and changes in supplemental oxygen therapy, performance of additional test and other as minor alterations.

### Analysis and statistics

Patient characteristics and alterations in treatment and disposition were analysed and reported using descriptive statistics. In order to determine the agreement between pheripheral venous and arterial blood gas results, the mean difference (MD) and 95% limits of agreement (95% LoA) were calculated using the Bland-Altman analysis. The SaO_2_ and the SpO_2_ were compared using the Passing-Bablok regression analysis and Bland-Altman analysis. Analyses were performed with IBM SPSS Statistics 26 and Analyse-it (Excel).

## Results

### Baseline characteristics

During the 12-week study period, we enrolled 157 patients. Three patients were excluded due to protocol violation (a prolonged time interval between ABG and pVBG sampling), leaving 154 patients for analysis (Table 1). Median age of those patients was 72 (IQR 62–80) years, and 67 (43.5%) were female. The most frequently documented comorbidity was COPD (n = 80, 51.9%) and 126 (81.8%) participants were current or ex-smokers.

**Table 1:** Baseline characteristics.

*Age (years), median (IQR)*	*72 (62–80)*
*Gender, n (%)*	
Male	87 (56.5%)
Female	67 (43.5%)
*Smoking status, n (%)*	
Non-smoker	28 (18.2%)
Ex-smoker	79 (51.3%)
Smoker	47 (30.5%)
*Comorbidities, n (%)*	
COPD	80 (51.9%)
Gold I	1 (1.2%)
Gold II	24 (30.0%)
Gold III	24 (30.0%)
Gold IV	20 (25.0%)
Unknown Gold classification	11 (13.8%)
Heart failure	34 (22.1%)
Asthma	36 (23.4%)
Charlson Comorbidity Index (CCI), mean (IQR)	4 (3–6)
*Recent (<30 days) hospital admission for similar complaints or diagnosis, n (%)*	*20 (13.0%)*
*Vital signs*	
Heart rate (bmp), median (IQR)	90 (78–106)
Systolic blood pressure (mmHg), median (IQR)	140 (123–155)
Diastolic blood pressure (mmHg), median (IQR)	78 (68–91)
Respiratory rate (per minute), median (IQR)	22 (20–25)
Temperature (°C), median (IQR)	37.4 (36.7–37.9)
Peripheral oxygen saturation (%), median (IQR)	93 (89–95)
Mental status (Glasgow Coma Scale), median (IQR)	15 (15–15)
*Specialty of the treating physician in the ED, n (%)*	
Emergency medicine	57 (37.0%)
Pulmonology	93 (60.4%)
Cardiology	2 (1.3%)
Internal medicine	2 (1.3%)
*Disposition, n (%)*	
Discharge from the ED	19 (12.3%)
Admission	135 (87.7%)
Admission to the ward	128 (94.8%)
Admission to the Cardiac Care Unit	5 (3.7%)
Admission to the Intensive Care Unit	2 (1.5%)
*Duration of hospital admission (days), median (IQR)*	*5 (3-7)*
*Mechanical ventilation, n (%)*	
Non-invasive ventilation	4 (2.6%)
High flow nasal oxygen therapy	1 (0.6%)
Intubation	0 (0.0%)
*Diagnosis at hospital discharge, n (%)*	
COPD exacerbation	61 (39.6%)
Heart failure	26 (16.9%)
Asthma exacerbation	18 (11.7%)
Upper respiratory tract infection	32 (20.8%)
Pulmonary embolism	3 (1.9%)
Pneumonia	52 (33.8%)
Other	43 (27.9%)
Combination of diagnosis	68 (44.2%)
*In-hospital mortality, n (%)*	*11 (7.1%)*

During blood gas sampling, 49 (31.8%) patients received supplemental oxygen therapy, with a median FiO_2_ of 28% (IQR 28–32%). Median time between venous and arterial blood gas sampling was three (IQR 2–4) minutes and a tourniquet was used during pVBG sampling in 24 (15.6%) patients.

### ABG versus pVBG: Treatment alterations

For 56 (36.4%) patients, the ABG results led to changed treatment and/or disposition, when compared to the pVBG results. Among these 56 patients, a total of 73 alterations occurred. The most frequent alteration was a change in supplemental oxygen therapy (n = 42, 57.5%) (Table 2).

**Table 2:** Alterations in treatment and disposition.

*Alteration of disposition, n (%)*	*11 (15.1%)*
Discharge instead of admission, n	3
Admission to the ward instead of discharge, n	5
Admission to the ward instead of the ICU, n	3
*Alteration of supplemental oxygen therapy, n (%)*	*42 (57.5%)*
Increasing, n	12
Decreasing, n	5
Starting, n	21
Stopping, n	4
*Alteration of mechanical ventilation, n (%)*	*7 (9.6%)*
Stop non-invasive ventilation, n	7
*Performing additional tests, n (%)*	*10 (13.7%)*
Imaging, n	3
Laboratory test, n	6
Consulting another specialty, n	1
*Other, n (%)*	*3 (4.1%)*
Withholding medication, n	3

For 31 (55.4%) patients, the alterations in treatment or disposition were caused by a single ABG value: for three (5.4%) patients by the pH, for six (10.7%) patients by the pCO_2_ and for 22 (39.3%) patients by the pO_2_ value. For the remaining 25 (44.6%) patients the alterations were caused by a combination of these values. No alterations in treatment or disposition were caused by the arterial bicarbonate or lactate values.

### Agreement between ABG, pVBG and pulse oximetry values

**Fig 1:**
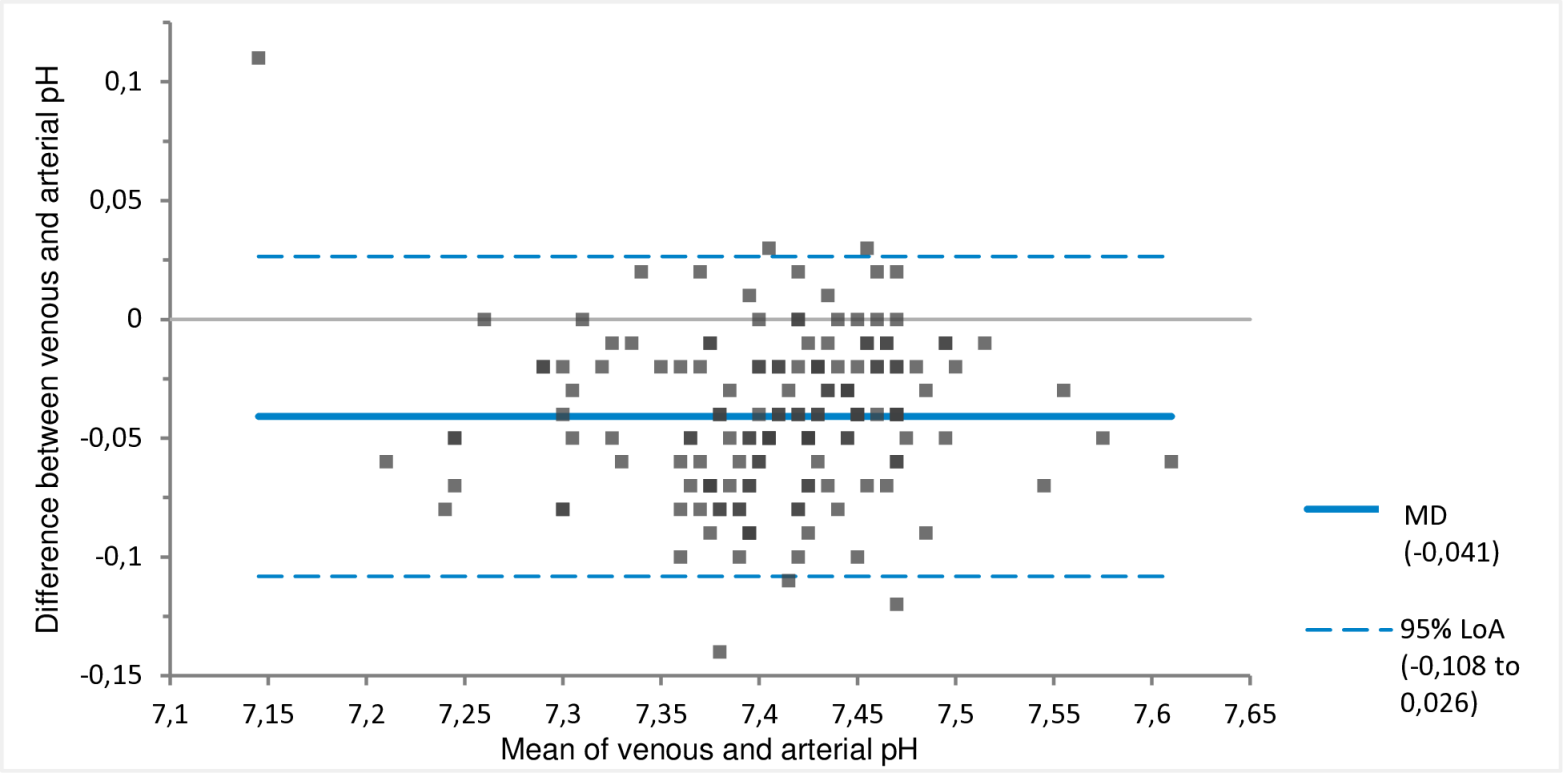
MD and 95% LoA for peripheral venous and arterial pH. Calculated using Bland-Altman analysis. MD = solid line. 95% LoA = dotted lines.

**Fig 2:**
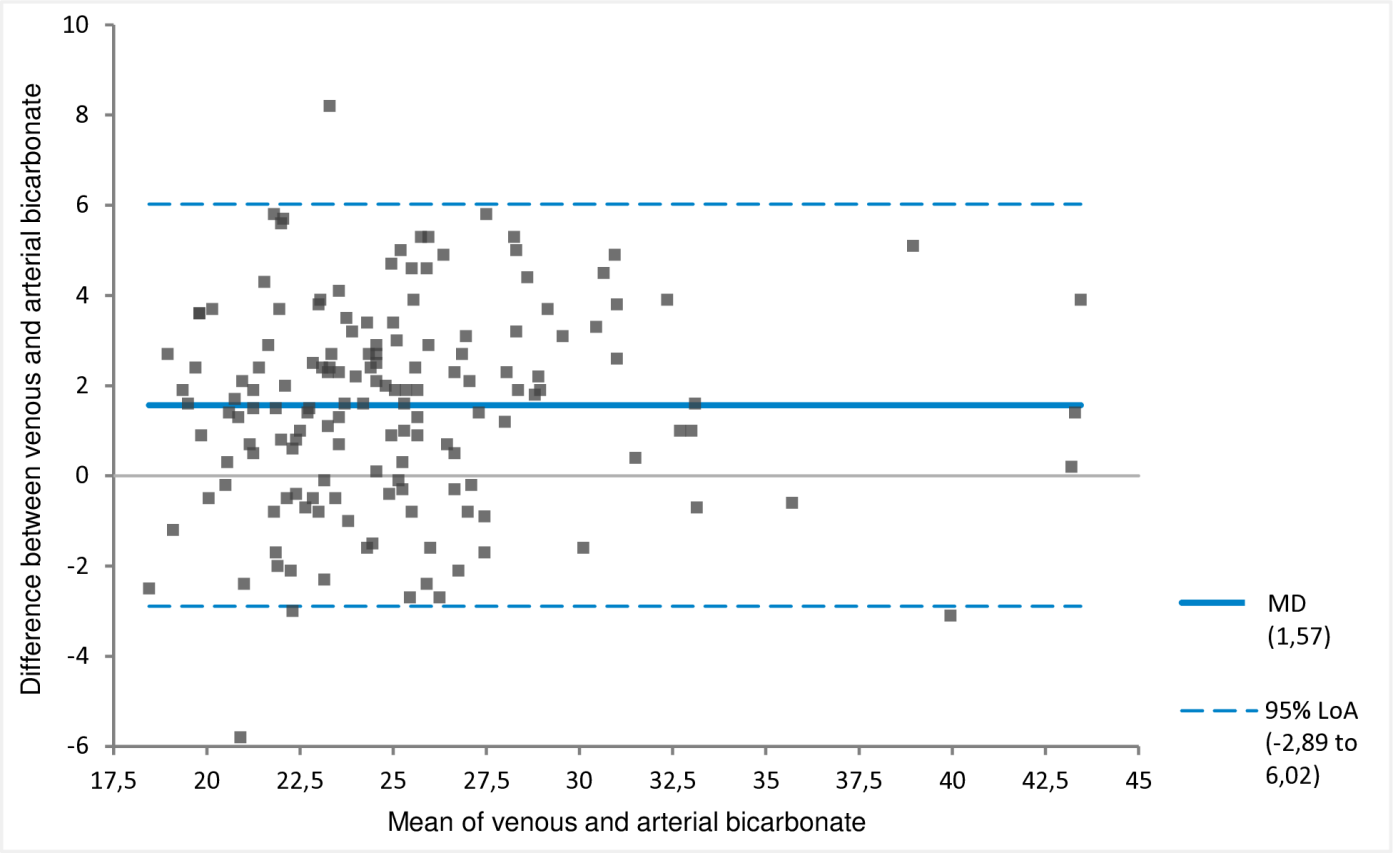
MD and 95% LoA for peripheral venous and arterial bicarbonate. Calculated using Bland-Altman analysis. Bicarbonate is displayed in mmol/l. MD = solid line. 95% LoA = dotted lines.

**Fig 3:**
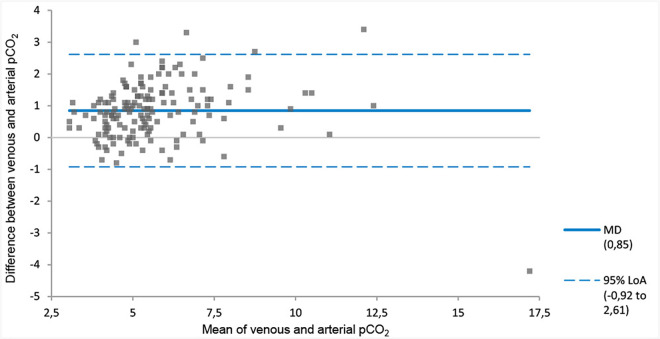
MD and 95% LoA for peripheral venous and arterial pCO_2_. Calculated using Bland-Altman analysis. pCO_2_ is displayed in kPa. MD = solid line. 95% LoA = dotted lines.

**Fig 4:**
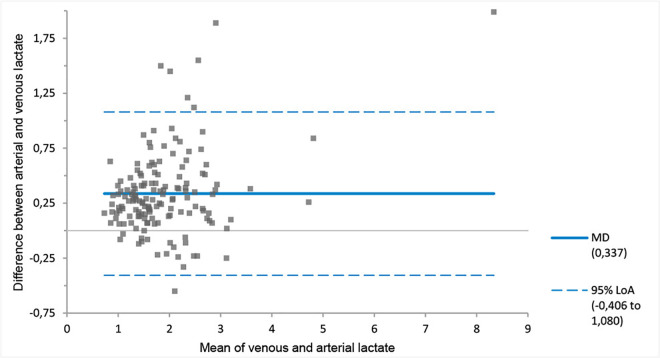
MD and 95% LoA for peripheral venous and arterial lactate. Calculated using Bland-Altman analysis. Lactate is displayed in mmol/l. MD = solid line. 95% LoA = dotted lines.

**Fig 5:**
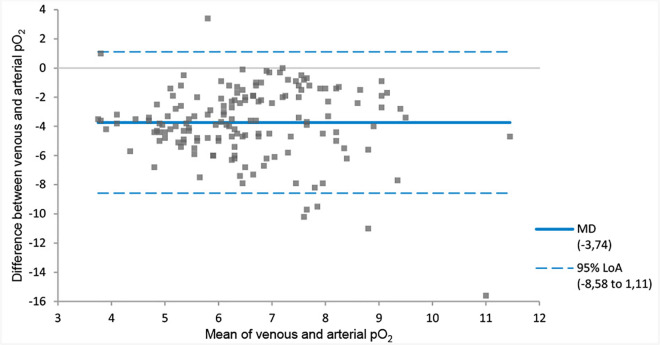
MD and 95% LoA for peripheral venous and arterial pO_2_. Calculated using Bland-Altman analysis. pO_2_ is displayed in kPa. MD = solid line. 95% LoA = dotted lines.

The MD between the venous and arterial pH of −0.04 (venous pH < arterial pH) with 95% LoA of −0.11 to 0.03 pH units (Fig 1, Table 3). On average, the venous bicarbonate, pCO_2_ and lactate values were higher than the arterial value (Fig 2–4, Table 3). The corresponding 95% LoA were respectively −2.89 to 6.02 mmol/l, −0.92 to 2.61 kPa and −0.41 to 1.08 mmol/l. The pO_2_ had the largest MD of −3.74 kPa (venous pO_2_ < arterial pO_2_) and widest 95% LoA of −8.58 to 1.11 kPa (Fig 5, Table 3). All 95% LoA include zero, which means the pVBG values were lower for some patients, but higher for other patients when compared to the ABG values.

**Table 3:** Agreement between arterial and peripheral venous pH, bicarbonate, pCO_2_, lactate and pO_2_.

Blood gas value	Total (n = 154)		No treatment alteration (n = 98)		Treatment alteration (n = 56)	
	MD* (95% CI)	95% LOA	MD* (95% CI)	95% LOA	MD* (95% CI)	95% LOA
pH	−0.04 (−0.05 to −0.04)	−0.11 to 0.03	−0.04 (−0.04 to −0.03)	−0.11 to 0.03	−0.05 (−0.06 to −0.04)	−0.11 to 0.01
Bicarbonate (mmol/l)	1.57 (1.20 to 1.93)	−2.89 to 6.02	1.30 (0.85 to 1.76)	−3.16 to 5.76	2.03 (1.43 to 2.62)	−2.32 to 6.38
pCO_2_ (kPa)	0.85 (0.70 to 0.99)	−0.92 to 2.61	0.69 (0.51 to 0.87)	−1.07 to 2.45	1.12 (0.89 to 1.34)	−0.55 to 2.78
Lactate (mmol/l)	0.34 (0.28 to 0.40)	−0.41 to 1.08	0.28 (0.20 to 0.35)	−0.43 to 0.99	0.44 (0.34 to 0.55)	−0.32 to 1.20
pO_2_ (kPa)	−3.74 (−4.13 to −3.34)	−8.58 to 1.11	−3.68 (−4.17 to −3.19)	−8.45 to 1.08	−3.83 (−4.52 to −3.15)	−8.85 to 1.18

* pVBG – ABG

We found no differences in agreement between the pVBG and ABG values when comparing the groups with and without treatment alterations (Table 3).

The mean of each ABG and pVBG value are provided in S1 Table in the supporting information.

**Fig 6:**
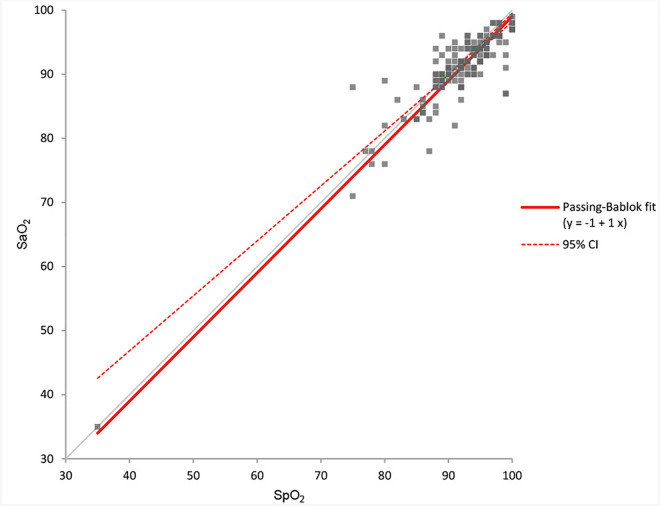
Correlation between SaO_2_ and SpO_2_. Calculated using Passing-Bablok regression analysis.

**Fig 7:**
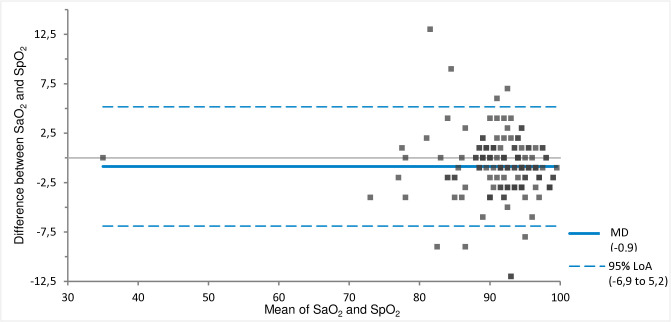
MD and 95% LoA for SaO_2_ and SpO_2_. Calculated using Bland-Altman analysis. MD = solid line. 95% LoA = dotted lines.

Comparing the SaO_2_ with the SpO_2_ measured with pulse oximetry showed a good correlation with an intercept of −1.0 (95% CI = −1.0 to 12.6) and a slope of 1.0 (95% CI = 0.9 to 1.0) (Fig 6). The MD was −0.9% (SpO_2_ < SaO_2_) with 95% LoA of −6.9 to 5.2% (Fig 7).

### Follow-up

In total, 135 (87.7%) patients were admitted to the hospital, most often (n = 61, 39.6%) with a COPD exacerbation. Five (3.2%) patients were treated with mechanical ventilation and 11 (7.1%) patients died during hospital stay (Table 1).

## Discussion

In this study, we investigated whether pVBG analysis combined with pulse oximetry could replace ABG analysis in the management of patients with undifferentiated respiratory complaints in the ED. We found that ABG results altered treatment for over one third of the patients and most alterations could be considered minor.

When comparing our results to previous studies, we found that only one other study previously examined the effect of ABG and pVBG analyses on clinical decision making. Among patients with DKA, ABG results rarely changed treatment and/or disposition [23]. Most likely, that contrast is due to the differences in study populations. For patients with respiratory complaints, the pO_2_, pCO_2_ and pH (i.e., the respiratory status) determine treatment and disposition, whereas for patients with DKA, the pH, bicarbonate and lactate (i.e., the metabolic status) are more important. The latter are less influenced by the gas exchange in peripheral tissues, therefore likely causing less alterations in treatment and admission. Another possible explanation could be that physicians are accustomed to using ABG results to determine treatment and disposition for patients with respiratory complaints. A subanalysis of our results showed that patients in which a change in treatment was initiated, more often were treated by pulmonologists than by emergency physicians (48.4% vs 15.8%, p = 0.000). A substantiated explanation for this phenomenon is lacking.

In addition to the number of changes, it is important to zoom in on the nature of these changes as well. We found that most (42/73, 57.5%) changes consisted of adjustment of supplemental oxygen therapy: increasing, decreasing, starting, or stopping therapy. Unfortunately, we did not investigate how large the adjustments were, and – although improbable – whether the changes impacted patient outcomes. Changes with a more likely clinical impact, such as change in disposition or change in mechanical ventilation, occurred less frequently (n = 18, 24.8%).

Arterial pO_2_ was most frequently mentioned as the reason for the alterations in treatment and disposition. Although this seems logical at first glance, our study also showed that the SpO_2_ measured by pulse oximetry had a good correlation with the SaO_2_. For only two of the 154 (1.3%) patients was the difference between SpO_2_ and SaO_2_ > 5%. That supports our hypothesis that the pVBG combined with pulse oximetry could be an alternative to an ABG and requires some habituation. A theoretical disadvantage of pulse oximetry is that possible hyperoxia cannot be ruled out, but this is no different from the situation of hospitalised patients, for whom supplemental oxygen therapy is commonly based on SpO_2_ measurements as well.

When zooming in on the agreement between the pVBG and ABG results, we found small MDs for pH, bicarbonate, pCO_2_ and lactate, indicating venous values could provide a reliable alternative (Table 3).

There is little research addressing what difference between the venous and arterial value may be acceptable to physicians. One previous study investigated what a clinically relevant difference between venous and arterial values for pH, bicarbonate and pCO_2_ would be according to 26 emergency physicians. On average that study showed 0.05, 3.5 mmol/l and 0.88 kPa for pH, bicarbonate and pCO_2_ [24]. The MDs found in our study fall within these limits (without taking the 95% LoA into account).

### Strengths and limitations

To our knowledge, this is the first study investigating not only the agreement between pVBG and ABG results, but also clinical decision making in ED patients with undifferentiated respiratory complaints. The results of this study can be of influence to a substantial proportion of ED patients.

In addition, we included the required number of patients in only 12 weeks, without missing data. ABG analysis was performed at the treating physician’s discretion, which is representative of daily practice. Finally, we had short interval times between drawing ABG and pVBG, and we made sure no treatment changes were initiated between pVBG and ABG sampling, increasing the validity of our results.

Naturally, our approach also has some drawbacks. First, it is a single centre study, which means that extrapolation of our results to other EDs must be done with caution. Not only can local characteristics of other EDs influence clinical decision making, patients may also be assessed by different physicians, who are more or less accustomed to using either ABG or pVBG analysis. Finally, it is theoretically possible that some of the 38 physicians treating 154 patients experienced a personal learning effect, although a median of three patients per physician makes this unlikely. In future studies, it would be interesting to investigate that personal learning effect, for instance by confronting participating physicians with the agreement between pO_2_ and SaO_2_.

Finally, it is worth mentioning that the most critically ill patients, with corresponding extremes in blood gas values, fell outside the scope of our study. We would claim that it is wise to always perform an ABG in this patient category.

## Conclusion

Due to the good degree of agreement between peripheral venous and arterial pH, pVBG combined with pulse oximetry could possibly replace ABG analysis for some patients with respiratory complaints in the ED, especially since it can be acquired more quickly. Although ABG results caused physicians to change treatment and/or disposition in over one third of the cases, most changes could be considered as minor. Future research should focus on the physicians’ personal learning effect and the effect of minor changes in treatment and disposition on patient outcome.

## Supporting information

S1 Table Mean of arterial and peripheral venous blood gas values. (DOCX)

S2 Dataset All anonymised data included in the analysis. (XLSX)

## References

[1] McKeever TM, Hearson G, Housley G, Reynolds C, Kinnear W, Harrison TW, et al. Using venous blood gas analysis in the assessment of COPD exacerbations: a prospective cohort study. Thorax. 2016;71(3):210–5. doi: 10.1136/thoraxjnl-2015-207573 26628461 PMC4789825

[2] Chauvin A, Javaud N, Ghazali A, Curac S, Altar A, Ali T, et al. Reducing pain by using venous blood gas instead of arterial blood gas (VEINART): a multicentre randomised controlled trial. Emerg Med J. 2020;37(12):756–61. doi: 10.1136/emermed-2019-209287 32759347

[3] van Exsel JAJM, Simons SO, Kramers C, Heijdra YF. When is a venous blood gas analysis sufficient in the emergency department?. Ned Tijdschr Geneeskd. 2017;161:D785. 28145212

[4] Dev SP, Hillmer MD, Ferri M. Videos in clinical medicine. Arterial puncture for blood gas analysis. N Engl J Med. 2011;364(5):e7. doi: 10.1056/NEJMvcm0803851 21288091

[5] Kelly A-M. Can VBG analysis replace ABG analysis in emergency care?. Emerg Med J. 2016;33(2):152–4. doi: 10.1136/emermed-2014-204326 25552544

[6] Kelly AM, McAlpine R, Kyle E. Venous pH can safely replace arterial pH in the initial evaluation of patients in the emergency department. Emerg Med J. 2001;18(5):340–2. doi: 10.1136/emj.18.5.340 11559602 PMC1725689

[7] Golub J, Gorenjak M, Pilinger EŽ, Lešnik A, Markota A. Comparison between arterial and peripheral-venous blood gases analysis in patients with dyspnoea and/or suspected acute respiratory failure. Eur J Intern Med. 2020;75:112–3. doi: 10.1016/j.ejim.2020.01.026 32061495

[8] Lim BL, Kelly A-M. A meta-analysis on the utility of peripheral venous blood gas analyses in exacerbations of chronic obstructive pulmonary disease in the emergency department. Eur J Emerg Med. 2010;17(5):246–8. doi: 10.1097/MEJ.0b013e328335622a 19996974

[9] McCanny P, Bennett K, Staunton P, McMahon G. Venous vs arterial blood gases in the assessment of patients presenting with an exacerbation of chronic obstructive pulmonary disease. Am J Emerg Med. 2012;30(6):896–900. doi: 10.1016/j.ajem.2011.06.011 21908141

[10] Kelly A-M. The case for venous rather than arterial blood gases in diabetic ketoacidosis. Emerg Med Australas. 2006;18(1):64–7. doi: 10.1111/j.1742-6723.2006.00803.x 16454777

[11] Zeserson E, Goodgame B, Hess JD, Schultz K, Hoon C, Lamb K, et al. Correlation of Venous Blood Gas and Pulse Oximetry With Arterial Blood Gas in the Undifferentiated Critically Ill Patient. J Intensive Care Med. 2018;33(3):176–81. doi: 10.1177/0885066616652597 27283009 PMC5885755

[12] Wong EKC, Lee PCS, Ansary S, Asha S, Wong KKH, Yee BJ, et al. Role of venous blood gases in hypercapnic respiratory failure chronic obstructive pulmonary disease patients presenting to the emergency department. Intern Med J. 2019;49(7):834–7. doi: 10.1111/imj.14186 30515940

[13] Evans L, Rhodes A, Alhazzani W, Antonelli M, Coopersmith CM, French C, et al. Surviving sepsis campaign: international guidelines for management of sepsis and septic shock 2021. Intensive Care Med. 2021;47(11):1181–247. doi: 10.1007/s00134-021-06506-y 34599691 PMC8486643

[14] Weimar Z, Smallwood N, Shao J, Chen XE, Moran TP, Khor YH. Arterial blood gas analysis or venous blood gas analysis for adult hospitalised patients with respiratory presentations: a systematic review. Intern Med J. 2024;54(9):1531–40. doi: 10.1111/imj.16438 38856155

[15] van Tienhoven AJ, van Beers CAJ, Siegert CEH. Agreement between arterial and peripheral venous lactate levels in the ED: A systematic review. Am J Emerg Med. 2019;37(4):746–50. doi: 10.1016/j.ajem.2019.01.034 30686538

[16] Bloom BM, Grundlingh J, Bestwick JP, Harris T. The role of venous blood gas in the emergency department: a systematic review and meta-analysis. Eur J Emerg Med. 2014;21(2):81–8. doi: 10.1097/MEJ.0b013e32836437cf 23903783

[17] Kelly A-M, Kyle E, McAlpine R. Venous pCO(2) and pH can be used to screen for significant hypercarbia in emergency patients with acute respiratory disease. J Emerg Med. 2002;22(1):15–9. doi: 10.1016/s0736-4679(01)00431-0 11809551

[18] Lee WW, Mayberry K, Crapo R, Jensen RL. The accuracy of pulse oximetry in the emergency department. Am J Emerg Med. 2000;18(4):427–31. doi: 10.1053/ajem.2000.7330 10919532

[19] Kelly AM, McAlpine R, Kyle E. How accurate are pulse oximeters in patients with acute exacerbations of chronic obstructive airways disease?. Respir Med. 2001;95(5):336–40. doi: 10.1053/rmed.2001.1046 11392573

[20] Garcia-Gutierrez S, Unzurrunzaga A, Arostegui I, Quintana JM, Pulido E, Gallardo MS, et al. The Use of Pulse Oximetry to Determine Hypoxemia in Acute Exacerbations of COPD. COPD. 2015;12(6):613–20. doi: 10.3109/15412555.2014.995291 25774875

[21] Timmers M, van Duijn D, Kompanje EJO. Is medical scientific research allowed in emergency situations without prior consent from the patient?. Ned Tijdschr Geneeskd. 2019;163:D3857. 31140767

[22] Charlson ME, Pompei P, Ales KL, MacKenzie CR. A new method of classifying prognostic comorbidity in longitudinal studies: development and validation. J Chronic Dis. 1987;40(5):373–83. doi: 10.1016/0021-9681(87)90171-8 3558716

[23] Ma OJ, Rush MD, Godfrey MM, Gaddis G. Arterial blood gas results rarely influence emergency physician management of patients with suspected diabetic ketoacidosis. Acad Emerg Med. 2003;10(8):836–41. doi: 10.1111/j.1553-2712.2003.tb00625.x 12896883

[24] Rang LCF, Murray HE, Wells GA, Macgougan CK. Can peripheral venous blood gases replace arterial blood gases in emergency department patients?. CJEM. 2002;4(1):7–15. doi: 10.1017/s1481803500006011 17637143

