# Supplementary material for: Comparison of peripheral venous and arterial blood gas in management of patients with respiratory complaints in the emergency department: A prospective observational cohort study
Source: PLoS One. 2025 Sep 5;20(9):e0330190. doi: 10.1371/journal.pone.0330190 (PMC12412979; doi:10.1371/journal.pone.0330190)
Supplement: S1 Table — (DOCX) [file pone.0330190.s001.docx]

| Blood gas value | Arterial blood gas | | | Peripheral venous blood gas | | |
| --- | --- | --- | --- | --- | --- | --- |
|  | Total (n=154), mean (95% CI) | No treatment alteration (n=98), mean (95% CI) | Treatment alteration (n=56), mean (95% CI) | Total (n=154), mean (95% CI) | No treatment alteration (n=98), mean (95% CI) | Treatment alteration (n=56), mean (95% CI) |
| pH | 7.43 (7.42-7.44) | 7.43 (7.41-7.44) | 7.43 (7.41-7.45) | 7.39 (7.38-7.40) | 7.39 (7.38-7.40) | 7.38 (7.36-7.40) |
| Bicarbonate (mmol/l) | 24.48 (23.75-25.20) | 24.24 (23.37-25.12) | 24.88 (23.58-26.18) | 26.04 (25.29-26.80) | 25.55 (24.63-26.47) | 26.91 (25.60-28.22) |
| pCO_2_ (kPa) | 5.23 (4.92-5.53) | 5.21 (4.80-5.62) | 5.25 (4.79-5.72) | 6.07 (5.76-6.38) | 5.90 (5.53-6.27) | 6.37 (5.81-6.93) |
| Lactate (mmol/l) | 1.75 (1.60-1.90) | 1.84 (1.62-2.05) | 1.60 (1.45-1.75) | 2.05 (1.90-2.20) | 2.06 (1.84-2.27) | 2.04 (1.86-2.21) |
| pO_2_ (kPa) | 8.42 (8.11-8.73) | 8.58 (8.24-8.93) | 8.13 (7.51-8.76) | 4.68 (4.40-4.96) | 4.90 (4.56-5.24) | 4.30 (3.82-4.78) |

**Table S1. Mean of arterial and peripheral venous blood gas values.**
