# Supplementary material for: Comparison of peripheral venous and arterial blood gas in management of patients with respiratory complaints in the emergency department: A prospective observational cohort study
Source: PLoS One. 2025 Sep 5;20(9):e0330190. doi: 10.1371/journal.pone.0330190 (PMC12412979; doi:10.1371/journal.pone.0330190)
Supplement: Attachment — Submitted filename: Response to Reviewers.docx [file pone.0330190.s004.docx]

13 July 2025

Subject: Revision of manuscript ‘Comparison of peripheral venous and arterial blood gas in management of patients with respiratory complaints in the emergency department: A prospective observational cohort study’

Dear Academic Editor,

We appreciate the time and effort you and each of the reviewers have dedicated to providing feedback and are pleased to have an opportunity to resubmit our revised manuscript ‘Comparison of peripheral venous and arterial blood gas in management of patients with respiratory complaints in the emergency department: A prospective observational cohort study’. In the revised manuscript, we have carefully considered reviewers’ comments and suggestions. We reply to each comment in point-by-point fashion.

**Journal requirements:**

1. Please ensure that your manuscript meets PLOS ONE's style requirements, including those for file naming. The PLOS ONE style templates can be found at https://journals.plos.org/plosone/s/file?id=wjVg/PLOSOne_formatting_sample_main_body.pdf and https://journals.plos.org/plosone/s/file?id=ba62/PLOSOne_formatting_sample_title_authors_affiliations.pdf

We made sure the manuscript meets PLOS ONE’s style requirements.

2. In the ethics statement in the Methods, you have specified that verbal consent was obtained. Please provide additional details regarding how this consent was documented and witnessed, and state whether this was approved by the IRB.

All eligible patients were approached for participation upon arrival in the ED and were given succinct verbal information about the study by the treating physician. After giving verbal consent to the treating physician to study participation the ABG and pVBG samples were collected. The verbal consent was documented in the Electronic Patient Record.

As soon as possible after initial treatment and stabilization, the patient or their representative received additional verbal and written information. Subsequently, written informed consent was obtained within 24 hours. Only after the written informed consent were data registered in the study database. If the patient died before giving written consent, the collected data were included in the study and the representative was informed of inclusion in the study.

The study was reviewed and approved by the local medical ethics committee (METC-Z2019070).

3. When completing the data availability statement of the submission form, you indicated that you will make your data available on acceptance. We strongly recommend all authors decide on a data sharing plan before acceptance, as the process can be lengthy and hold up publication timelines. Please note that, though access restrictions are acceptable now, your entire data will need to be made freely accessible if your manuscript is accepted for publication. This policy applies to all data except where public deposition would breach compliance with the protocol approved by your research ethics board. If you are unable to adhere to our open data policy, please kindly revise your statement to explain your reasoning and we will seek the editor's input on an exemption. Please be assured that, once you have provided your new statement, the assessment of your exemption will not hold up the peer review process.

Our dataset will be available as a Supplement to our article. All co-authors agreed in advance with the data sharing plan.

4. Please ensure that you refer to Figures 1-6 in your text as, if accepted, production will need this reference to link the reader to the figure.

We refer to Figures 1-6 in our text.

5. Please review your reference list to ensure that it is complete and correct. If you have cited papers that have been retracted, please include the rationale for doing so in the manuscript text, or remove these references and replace them with relevant current references. Any changes to the reference list should be mentioned in the rebuttal letter that accompanies your revised manuscript. If you need to cite a retracted article, indicate the article’s retracted status in the References list and also include a citation and full reference for the retraction notice.

We reviewed our references list. No articles were retracted.

We added three articles suggested by reviewer #3 to the references list.

- Golub J, Gorenjak M, Pilinger E, Lešnik A, Markota A. Comparison between arterial and peripheral-venous blood gases analysis in patients with dyspnoea and/or suspected acute respiratory failure. European journal of internal medicine. 2020;75:112-3 <https://doi.org/10.1016/j.ejim.2020.01.026>.
- Weimar Z, Smallwood N, Shao J, Chen XE, Moran TP, Khor YH. Arterial blood gas analysis or venous blood gas analysis for adult hospitalised patients with respiratory presentations: a systematic review. Internal medicine journal. 2024;54(9):1531-40 <https://doi.org/10.1111/imj.16438>.
- Wong EKC, Lee PCS, Ansary S, Asha S, Wong KKH, Yee BJ, Ng AT. Role of venous blood gases in hypercapnic respiratory failure chronic obstructive pulmonary disease patients presenting to the emergency department. Intern Med J. 2019 Jul;49(7):834-837. doi: 10.1111/imj.14186. PMID: 30515940.

**Review Comments to the Author:**

*Reviewer #1:*

Comments

1. I commend the authors on the eloquent design and execution of their study.

2. Though beyond the scope of their original design, as they have the raw data, I would be curious to know if larger differences in oxygen saturation and / or the partial pressure of carbon dioxide between the paired pVBG and ABG, were associated with severity of illness and or final patient outcome, including mortality.

Thank you for your compliment and reviewing our article.

We do agree it would be interesting to know if larger differences in oxygen saturation and/or the partial pressure of carbon dioxide between the paired pVBG and ABG, were associated with severity of illness and or final patient outcome. Unfortunately, we do not have enough data on severity of illness and final patient outcome to answer this question. Hopefully, future research will focus on these study endpoints.

*Reviewer #2:*

This is an interesting study, and the manuscript is well-written. However, I have one concern regarding the primary endpoint - the decision to alter treatment or disposition. This decision may rely on clinicians' judgment, which can be subjective rather than objective. Please discuss this limitation.

Thank you for reviewing our article. You have raised an interesting point. We decided to rely on the clinicians’ judgement since it is the best representation of daily practice. That is why we think it is a strength rather than a limitation of our study.

*Reviewer #3:*

GENERAL COMMENTS

This is an interesting clinically relevant study addressing an important question, ie whether VBGs could be used instead of ABGs in assessing patients with respiratory complains, mainly exacerbated COPD or respiratory failure.

The main question of the study was whether pVBG analysis combined with pulse oximetry could replace ABG analysis to determine treatment and disposition of patients with undifferentiated respiratory complaints in the ED.

It is my understanding that the answer to this question is positive (consistent with previous similar studies reported below in the REFERENCES section). If so, the conclusions must be better explained, particularly focusing on what this paper add to previously known information (also reported in few selected papers published in the last 5 years reported below.

Thank you for reviewing our article.

We do not entirely agree with your conclusion that pVBG analysis combined with pulse oximetry could replace ABG analysis to determine treatment and disposition of some patients with undifferentiated respiratory complaints in the ED. In over one third of patients ABG results caused physicians to change treatment and/or disposition. Despite most changes could be considered as minor, it is still a relatively large proportion and no data on patient outcome exists.

However, outside of the scoop of our article, we could envision one might use a pVBG combined with pulse oximetry as a screening for acidosis, hypercapnia and hypoxemia. Especially if no qualified nurse of physician is available to collect an ABG in a crowded Emergency Department.

Until data on patient outcome will be available, we believe ABG remains the reference standard test.

SPECIFIC COMMENTS

In order to make the paper more readable, I would recommend to include in the tables both the absolute values of each parameter in addition to the differences.

Thank you for this suggestion. To maintain the clarity and structure of our article and Table 3, we added a table with the absolute values of each blood gas value as a supplement to the article.

SELECTED REFERENCES 2020-2025 ON VBG VS ABG

1: Weimar Z, Smallwood N, Shao J, Chen XE, Moran TP, Khor YH. Arterial blood gas analysis or venous blood gas analysis for adult hospitalised patients with respiratory presentations: a systematic review. Intern Med J. 2024 Sep;54(9):1531-1540. doi: 10.1111/imj.16438. Epub 2024 Jun 10. PMID: 38856155.

2: Lindstrom SJ, McDonald CF, Howard ME, O'Donoghue FJ, McMahon MA, Rautela L, Churchward T, Biesenbach P, Rochford PD. Venous blood gases in the assessment of respiratory failure in patients undergoing sleep studies: a randomized study. J Clin Sleep Med. 2024 Aug 1;20(8):1259-1266. doi: 10.5664/jcsm.11128. PMID: 38525926; PMCID: PMC11294137.

3: Davies MG, Wozniak DR, Quinnell TG, Palas E, George S, Huang Y, Jayasekara R, Stoneman V, Smith IE, Thomsen LP, Rees SE. Comparison of mathematically arterialised venous blood gas sampling with arterial, capillary, and venous sampling in adult patients with hypercapnic respiratory failure: a single-centre longitudinal cohort study. BMJ Open Respir Res. 2023 Jun;10(1):e001537. doi: 10.1136/bmjresp-2022-001537. PMID: 37369550; PMCID: PMC10335414.

4: Golub J, Gorenjak M, Pilinger EŽ, Lešnik A, Markota A. Comparison between arterial and peripheral-venous blood gases analysis in patients with dyspnoea and/or suspected acute respiratory failure. Eur J Intern Med. 2020 May;75:112-113. doi: 10.1016/j.ejim.2020.01.026. Epub 2020 Feb 12. PMID: 32061495.

5: Byrne AL, Bennett MH, Chatterji R, Symons R, Thomas PS. Arterial and venous blood gases in exacerbations of chronic obstructive pulmonar disease. Intern Med J. 2020 Jan;50(1):133-134. doi: 10.1111/imj.14692. PMID: 31943620.

6: Wong EKC, Lee PCS, Ansary S, Asha S, Wong KKH, Yee BJ, Ng AT. Role of venous blood gases in hypercapnic respiratory failure chronic obstructive pulmonary disease patients presenting to the emergency department. Intern Med J. 2019 Jul;49(7):834-837. doi: 10.1111/imj.14186. PMID: 30515940.

Thank you for suggesting additional references. We added three suggested articles (Weimar et al., Golub et al., Wong et al.) as references to our article.

Earlier in our research, we came across the article of Byrne et al. We decided not to add this article to our reference list, because of the more recent systematic review of Weimar et al.

The articles of Lindstrom et al. and Davies et al. are interesting and show pVBG can be used in multiple settings and different ways, however the setting of these articles is beyond the scope of our study.
